# Rubber Work

**Published:** 1861-11

**Authors:** 


### RUBBER WORK.

We are often asked, "Do you still use rubber work," to which we reply we do, and hope to continue to do so; indeed we could not well get along without it. There are cases for which it is far better than anything else. We do not now propose to specify the peculiarities of those cases for which it is especially adapted; that may be done hereafter.

We use gold, continuous gum and rubber work in nearly equal proportions. When a case is presented, the operator should examine and decide what would be best, and give the patient the result of his judgment; in this, of course, the patient should acquiesce, if there are no considerations rendering a different course necessary.

There are two or three points in reference to rubber work, about which little or nothing has been said, that are worthy of attention. One is in regard to vulcanizing pieces of different thickness: the thick require considerable more time in the vulcanizer, under the same degree of heat, than a thin piece. This will be governed by the thickness of the piece, of course, and should be closely observed by the workman.

There is a very common opinion that rubber work can not be repaired when broken, or it is comparatively worthless if it is repaired; this is certainly not correct, for we find almost every kind of breakage as easily mended in rubber work as any other, even to the splitting in two of an entire plate—which, however, if the work is well done, will not occur—such we have seen and repaired, too, making them quite as good as before broken.

Teeth that are broken off may be replaced, and fractures mended, about as well as with any other kind of work. The most difficult pieces to mend are those that are very thin at, and in the vicinity of the fracture; but there is little difficulty with them; little grooves may be cut from the fractured edges into the plate, or little holes may be made through the plate a short distance back from the fractured edge; then the rubber may be made to overlap the entire fracture, passing through the grooves or holes, and just make the piece as firm as ever. It will add something to the thickness; this, however, is the case with any kind of mending.

But the facility with which this work may be mounted, renders the mending in many cases less imperative than the other styles of work. The teeth can be removed without injury, and remounted as perfectly as at first; and more than this, if an impression of the mouth can not be obtained, the old piece may be used as a model from which to build the new, if there is not too much change in the mouth of the patient. This would only be necessary in extreme cases. The objection is made to rubber work, that so few dentists make it, that difficulty is experienced in getting it repaired or changed except in the cities. The reply to this is, that if rubber work is good, every dentist should be in possession of the process. A dentist remarked recently, "Well, I think there is nothing so good for all cases as gold, and so I will stick to the old stand-by; I don't like new things any way, they are generally humbugs." We have about the same respect for the opinions of the man who distrusts and refuses every thing, because it is new, and tenaciously adheres to an old thing, or process, because in some cases it is good, and he knows its ways and tricks; as for the opinion of him who discards everything because it is old, and mounts every new thing that comes along, regardless of its merits, and rides it, hobby-like, with a zeal that knows no bounds, until he is thrown off, or till some newer thing comes along, when he dismounts and mounts again. The true course is to select that which is valuable, whether new or old, and use it. T.

